# Capturing the Elite in Marine Conservation in Northeast Kalimantan

**DOI:** 10.1007/s10745-016-9830-0

**Published:** 2016-06-24

**Authors:** Rini Kusumawati, Leontine Visser

**Affiliations:** Bogor Agricultural University (IPB), Bogor, Jawa Barat Indonesia; Wageningen University, Wageningen, Netherlands

**Keywords:** Elite capture, Networks, Marine conservation, Kalimantan

## Abstract

This article takes the existence of power networks of local elites as a social fact of fundamental importance and the starting point for the study of patronage in the governance of the coastal waters of East Kalimantan. We address the question of how to capture the elites for project implementation, rather than assuming the inevitability of elite capture of project funds. We analyze the multiple-scale networks of local power holders (*punggawa*) and the collaboration and friction between the political-economic interests and historical values of local actors and the scientific motivations of international environmental organizations. We describe how collaboration and friction between members of the elite challenge models that categorically exclude or co-opt local elites in foreign projects. In-depth ethnographic study of these networks shows their resilience through flows of knowledge and power in a highly volatile coastal environment. Results indicate the need for inclusion in decision making of local entrepreneurs, and – indirectly - their dependents in decentralized coastal governance.

## Introduction

### Patrons, Clients and the Governance of Local Elites

There exists an almost universal social mechanism that gives a historical elite a prominent role in society (Esman and Uphoff 1984: 249). In the Netherlands-Indies, the Dutch administrators quickly grasped the potential of the Javanese *priyayi* elite by giving practical training to increase their bureaucratic expertise and skills, but within as much of an indigenous milieu as possible. The Dutch were the patrons, the indigenous elite the clients (Sutherland 1979: 45, 53). A patron–client relationship was purposely seen by the Dutch as a one-dimensional administrative order (ibid.). This colonial view sharply contrasted with the multiple forms of authority that existed then and now (Warren 1993) between local elites as patrons and their dependents; orders of relations through multiple-scale networks that may be social, economic, political and cultural – often all at the same time.

Although certainly not restricted to Indonesia, the strongly hierarchical Buginese and Makassarese societies of southern Sulawesi provide well-known examples of patron–client relationships (Sutherland 2001; Acciaioli 2000; Pelras 2000; Pelras 1996). In the English translation (Chabot 1996) of his 1950 PhD dissertation Chabot already shows the historical value and the multiple social roles of the patron as landowner, merchant, and governor in his relationships to clients (Chabot 1996: 149–158). Pelras’ ethnographic research of the 1960s and 1970s elaborates on the political aspects of the patronage of noblemen and the ways they are able to extend their clientele. Although the administrative role of nobility as *punggawa* dwindled during the last decades of the twentieth century, they still constitute part of the new elite, and patron–client ties prove to be remarkably resilient not only in political, but increasingly also in economic terms (Pelras 2000).

The patron–client relationship can be characterized generally as an unequal (but theoretically nonbinding) relationship between a superior (a patron or leader) and a number of inferiors (clients, retainers or followers), based on an asymmetric exchange of services, where the de facto dependence on the patron of the clients, whose unpaid services may include economic obligations, paid or unpaid work, armed service, political support and other services, is counterbalanced by the role the patron plays as a leading figure for all the clients and by the assistance, including monetary loans and protection, he or she provides when necessary. (Pelras 2000: 16)

Acciaioli (2000) makes a clear distinction between the fish entrepreneur or *bos* in Lindu, Sulawesi from a patron (*punggawa*) on the grounds that the former is a leader in the narrowly economic sense. But in Lindu society the Bugis are a minority of migrants (Acciaioli 2000: 224), whereas in our case the Bugis on the coasts of northeast Kalimantan, including Berau, have established their own settlements as fishers and traders, in the virtual absence of more land-oriented Dayak villages. This may account for the fact that pervasive *punggawa* networks are still very much present in the Berau coastal area among the Buginese, Makassarese, Mandarese, and Bajau fishers and traders (Gunawan 2012; Kusumawati 2014; Pauwelussen 2015).

We regard patronage as a network emerging in the everyday life interactions of *punggawa* (patrons, leaders, traders, land/resource owners) and their clients, rather than as a one-dimensional hierarchical structure of a patron and his dependents (Chabot 1996; Pelras 1996, 2000). Both the patron and his/her clients are embedded in multiple and multi-scalar social, economic, political and cultural relationships that make them inter-dependent, be it in a highly dynamic context of shifting powers and moving resources (Gunawan and Visser 2012; Kusumawati and Visser 2014; Pauwelussen 2015). Moreover, patrons themselves may be the client of another, higher-ranking patron in a chain of political and economic interdependencies, in which power is not fixed but increases or dwindles through time and space.

The integral social, political-economic and cultural quality of the goods exchanged in patron–client relationships can be found all over Indonesia, from Java to West Papua, where ritual cloth (*kain timur*), land, and ‘development’ are exchanged between local elite families and the government (Visser 1999). Here too, elites develop through historical interaction with outside powers, as the Papuan elite of the late twentieth century were the sons and grandsons of local war lords and ritual leaders who were the leaders and teachers in pre-colonial Papuan society (Visser 2001). They were sent to school by the Dutch administration (1950–62) and the best graduates were selected to become indigenous government officials in the 1950s and the trusted brokers of development (Olivier de Sardan 2005; Visser 2012).

The Berau district in northeast Kalimantan covers 34,127 km^2^ of which 35.7 % are coastal waters (BPS Berau 2011). It is rich in forestry, mining and fishery resources (Obidzinski and Barr 2003). Since 1999 the implementation of decentralization in Indonesia (Laws 22/1999 and 25/1999, and Law No. 32/2004) has resulted in a greater role for the district government, particularly of resource-rich districts like Berau, in managing their natural resources (Resosudarmo 2004) and gaining more political power through governing these resources (Satria et al. 2006; Hidayat 2005; Hadiz 2004). The laws mandated that up to 80 % of natural resource revenues be re-directed to regional governments, rather than the 20 % prior to 1999 (Patlis 2008; Patlis 2005).

In Buginese-Makassarese societies in southern Sulawesi and eastern Kalimantan, the tendency of members of the local political elite to exhibit a ‘mono-centric-loyalty’ (Hidayat 2000) to their superior or boss (*punggawa*) or to other members of the local elite, rather than to the community as a whole, further strengthens the power of the district government. Today, the sovereign positioning of the district’s political elite implies that in every process of decision-making, priority is given to protecting their short-term political-economic interests over longer-term social-economic and cultural interests of the coastal communities.

In addition to their local and regional networks within Indonesia the local elite in Berau hold long-term international connections with patrons and Chinese traders (*tauke*) in Malaysia (Casson and Obidzinski 2002; Pauwelussen 2015). Interestingly, in their position as government officials and private entrepreneurs, several *punggawa* have become involved with global environmental organizations in the debate over and the final establishment of the Berau Marine Conservation Area in 2005 (Kusumawati 2014).

In this contribution to the volume we analyze patronage networks as the property of newly emerging social, economic, political and cultural relationships between coastal Buginese, Butonese, Makassarese, Mandarese, Bajau and other actors. We will focus on the political-economic role and position of the governmental and entrepreneurial elite in their involvement with foreign actors, in particular with the global environmental NGOs, in the establishment of the marine conservation area in Berau^1^. We do not see these networks primarily as phenomena of the technological revolution and the global political-economic information flows of a new economy as does Castells (2010), although the recent availability and widespread use of mobile phones, even in the coastal space of Kalimantan, does indeed contribute to strengthening existing *punggawa* networks. Instead, we focus on the way these networks constitute and are constituted from the perspective of the everyday practice of marine conservation in coastal northeast Kalimantan, although we do not deny that they are part of a global economy.

Patron–client relationships, although clearly hierarchical, create interdependency based on ‘friendship’, kinship, and alliance; patron–client commitments that are often enduring. While clients are clearly kept in debt dependencies (Acciaioli 2000; Gunawan 2012), patrons also depend on their clients for cheap labor, resource delivery, and political support (Kusumawati et al. 2013). Ever since the early research by Pelras since 1976 successive anthropological studies have documented well the regional and transnational networks of generations of *punggawa* who appear to be quite resilient, shifting their influence and dominance over time from one ‘frontier’ resource to another. Patrons as leaders and traders often extend their networks over wide social and geographical scales by skillfully manipulating their relationships within Indonesia and beyond. For example, the Berau elites have been documented to have moved from shipping and smuggling weapons across the border into Malaysia in the 1960s to illegal logging in the 1980–90s, long-term involvement in the turtle egg trade and, more recently, in the era of decentralization, shrimp aquaculture, mining and oil palm plantation, cyanide fisheries, and the transnational trade of live reef fish and giant clams (Casson and Obidzinski 2002; Obidzinski 2003; Gunawan 2012; Kusumawati et al. 2013; Kusumawati 2014; Schwerdtner Máñez and Pauwelussen 2014; Pauwelussen 2015)**.**

Rather than assuming that Berau government officials and private entrepreneurs negatively influence MCA establishment, we favor an approach that helps to understand how these actors who form the more advantaged class or elite might be ‘captured’ themselves in their relationships with global environmental actors. We think it is more relevant to study how patrons use their power in decision-making instead of assuming that they misuse their power in capturing the benefits of external projects solely for personal advantage. Moreover, we want to show how local elites themselves also become dependents of foreign powers. Our concern in this case is not the wise use of global donor funding, but the performance of members of local political, administrative and economic elites in the decision-making process about marine conservation, and their impact on the effectiveness of the implementation of the Berau MCA.

Alatas et al. (2013) correctly conclude that the assumption of elite capture by foreign projects results in the marginalization of the role of local leaders and their “long-term effects on institutional and bureaucratic performance” (Alatas et al. 2013: 2). We also concur with the now widely accepted observation (Wolf 1990) that understanding power is vital in approaching the problem of elite capture. Likewise, understanding *punggawa* networking is necessary to see how an external project like the establishment of the Berau MCA might ‘capture the elite’ and win them to the side of the international NGOs by taking the multiple social, economic, cultural and political interests of the Berau elite seriously. With this, we mean to study their performance to understand their power position and the lack of effectiveness of the Berau MCA.

Several authors have addressed the issue of inclusion or exclusion of local elites. Working at the University of Bradford, England, Sam Wong participated in a UNU-WIDER project on the role of elites in economic development to compare various so-called counter-elite and co-opt elite approaches of international donor-driven projects. By comparing these two approaches in two case studies in Bangladesh and Ghana respectively, Wong addresses the paradox of inclusion and exclusion of elites, and stresses the importance of breaking the dependency relationships of the poor in patron–client networks (Wong 2010: 2). Similarly, Labonte (2011) studying the co-opting of local chiefs in international projects in Sierra Leone, looks for a ‘solution’ proposing to mitigate the effect of elite capture by addressing the root of their power, namely rules about access to resources that keep the non-elite dependent. We take the position that inclusion of the private sector, such as the local entrepreneurial elite, is as important as the inclusion of the governmental actors in constructing a governance model linking local views about extraction-cum-conservation and global conservation demands.

## Methodological Considerations

This case study focuses on the culturally and historically strong position of local economic and political elites, and their role in the implementation of a marine conservation area (MCA) in the Berau district of East Kalimantan. International environmental NGOs, particularly The Nature Conservancy (TNC) and The Worldwide Fund for Nature (WWF), have opted for a selective inclusion of local governmental actors and the exclusion of a historically powerful political-economic elite (*punggawa*) from the decision-making process and implementation of the Berau MCA (Kusumawati 2014). This study of the internal dynamics of the local elite thus challenges simplistic models of exclusion (circumventing) or inclusion (co-opting) of local elites in project interventions (Wong 2010).

Empirical research studying the historical underpinning and the versatility of the everyday interactions in the networking of elites is an appropriate method to overcome what Wong states as the common weakness of both exclusion and inclusion approaches: an inadequate understanding of the interactions between elites and non-elites (Wong 2010: 5). He concludes that: “[T]he ‘counter-elite’ and ‘co-opt-elite’ approaches should not be seen as ‘either-or’. Elites can be absorbed and challenged in the same project at the same time” (Wong 2010: 15). Although we agree in general, this conclusion unfortunately does not bring us any closer to answering the question of *how* elites are captured (historically, socially, culturally, economically, politically, at multiple scales, and differently through time) in order for interventionists to effectively ‘capture’ the elites. Hence, in this paper we address that very question of ‘how to capture the elite?’ We analyze the multiple-scale networks of local power holders (*punggawa*) and the collaboration and friction between the political-economic interests in coastal resources of government agencies, private entrepreneurs, and international environmental organizations.

In-depth and long-term ethnographic research by the first author on these *punggawa* networks (Kusumawati 2014) shows their social resilience through flows of knowledge and power in a highly volatile coastal environment. It also shows that the concept of ‘local elite’ is too shallow to uncover the internal dynamics of the relationships between its members, both in terms of collaboration and friction. Primary data was gathered during field research between 2008 and 2011 (Kusumawati 2014) by observing and conducting in-depth interviews with multiple-scale actors involved in the development process of the marine conservation area, including national and regional government officials, local entrepreneurs, and international and local NGO staff. Interviews focused on the governance of the coastal area of Berau, the development process of the Berau MCA and the history of sea turtle management in Berau. Observations included attendance at meetings and workshops held by the environmental NGOs in their communication (*sosialisasi*) of the Berau Marine Conservation Area program to the district government. Secondary data to complement the empirical data was gathered by reviewing and examining a variety of secondary sources on the Berau MCA and on sea turtle egg exploitation from websites, newsletters, newspaper articles and reports produced by NGOs (Kusumawati and Visser 2014; Kusumawati et al. 2013)*.*

### Elite Capture or Capturing the Elite?

Elite capture is defined as the capture of public resources by local elites holding social, economic and political power (Chowdhury and Yamauchi 2010). International development organizations often assume that local elites appropriate or ‘capture’ a disproportionate part of project revenues or opportunities, resulting in the failure of development programs implemented by international donors together with national governments and NGOs, because of a lack of accountability and transparency of project organization (Platteau 2004). As a result, involvement of members of the local political-economic elite in donor-funded development programs is often seen as problematic. The issue has stimulated a debate on the inclusion or exclusion of elites from project interventions. In this section we address two major concerns with the conceptualization of elite capture: the international donors’ concern with financial effectiveness, and the institutional implications of project intervention overlooking the multiplicity of roles and positions of local elites (*punggawa*) as patrons of a wider ‘community’.

The World Bank and other international donor organizations have for about two decades been concerned with the financial effectiveness of the implementation of internationally funded programs involving community participation (Platteau 2004). Platteau’s research has in many ways set the tone for the introduction of the concept of ‘elite capture’ in terms of the misappropriation of project funds by local elites in community-driven development (Dutta 2009). The formulation of the problem determines its solution. Once elite capture is being formulated as the problem of local elites frustrating the effectiveness of international development interventions at the expense of the targeted ‘poor communities’, this practice needs to be mitigated. For example by conditional funds release, a more active role for ‘outsiders’ in project interventions, and by fraud detection mechanisms, but also by ‘disciplining’ the local elites (Platteau 2004). Interestingly, these ‘solutions’ for project interventions targeting poverty alleviation and democratic resource distribution seem oblivious of the societal relevance of the often historical and institutional embeddedness of multiple social, economic and political networks connecting the elites in the public and private domains of most of the so-called weak states where interventions by the United Nations, the International Monetary Fund, and the World Bank, or The Nature Conservancy and other global and national environmental NGOs are taking place.

In other words, local elites are captured in multiple networks where they are invariably the patron or the client. Moreover, the strength of the network lies in the fact that it involves social, economic and political relationships *at the same time*, and that the one tie supports (or weakens) the other. Regarding the local elite as captured within a political-economic network with strong cultural and historical roots thus demands a different approach to what has been labeled as ‘elite capture’. Elite capture is seen as a problem particularly in studies on community-driven development (Dasgupta and Beard 2007; Saito-Jensen et al. 2010; Wong 2010; Labonte 2011). Dasgupta and Beard (2007: 232) refer to several studies on the New Order regime of Soeharto (between 1965 to 1998) in Indonesia and recent local politics in Java and Sumatra, and cite Sidel’s conclusion (2004: 69–70) that “economic and political power differs from that of ‘local bosses’ in The Philippines and Thailand in that it is not being consolidated in the hands of individual strongmen or ‘dynasties’. At the regency, municipal, and provincial levels in Indonesia economic and political power appears to be associated with loosely defined, somewhat shadowy, and rather fluid clusters and cliques of businessmen, politicians and officials”, and “power-sharing arrangements, contestation between rival families and factions, and high turnover appear to be the norm”. They found that communities where both non-elites and elites participated in democratic self-governance indeed demonstrated an ability to redress capture when it occurred. A survey of 250 Community-Driven Development (CDD) projects in Indonesia (Fritzen 2007) shows that, although the participation of non-elites is on the rise, elites’ control of decision-making is still pervasive (see also Lucas, this volume).

We feel that such studies remain too much driven by a northern, hegemonic view and expatriate concern with the institutional norm of a Weberian transparent, democratic, and inclusive, but narrowly defined financial accountability, the resonance of which we find in the earlier work of institutionalists. Recently such structural–institutional approaches to project intervention have been criticized (Cleaver 2012; Li 2007). When a normative global concept like elite capture is imposed on an Indonesian reality, the outcome may be a definition like the following: “Local elites are locally based individuals with disproportionate access to social, political or economic power. The term elite capture refers to the process by which these individuals dominate and corrupt community-level planning and governance” (Dasgupta and Beard 2007: 230, note 1; see also Dutta 2009). This definition is based on an extensive literature review that seems to keep repeating these pejorative or prejudiced terms and to primarily address the negative aspects of local elite participation. But it disregards the multiple-scale character of the power networks of ‘local’ elites, as well as the connectivity of their social, cultural, economic and political powers performing as leaders, patrons and bosses to attract and redistribute^2^ external resources among their dependents (see Steenbergen, this volume). We are convinced that studies of elite capture should be based on in-depth empirical research over a longer period of time, and intimate knowledge of local power relationships (Wolf 1990; Lund and Saito-Jensen 2013). Unfortunately, international project staff are usually not in a position to register and value such long-term social processes. But ethnographic studies do and their empirical observations may be used to critically discuss the legitimacy or fairness and applicability of a global concept like elite capture to local conditions (McCarthy et al. 2014).

Another problem is the gloss of elite capture itself. Although observably individual actors and elites are mostly treated as a collective entity, ‘the local elite’ is certainly not the homogenous social category that is often described as the subject of much elite capture literature (Chowdhury and Yamauchi 2010). They are indeed members of a particular social class, and they do act as the representative of a collectivity, but along lines of extended families, trans-local kinship networks, and public–private exchange relationships in which one individual member of the elite is himself interdependent with others, within or outside the local community, including both clients and other patrons (Pauwelussen 2015; Gunawan and Visser 2012; Visser 2001).

### Local Elites’ Resource Management and Berau Marine Conservation Area

In this section, we will discuss the political and economic roles of the local elite in managing marine resources, especially the exploitation and trade of sea turtle eggs. This elite is actively engaged within district and village level political networks of Berau government, where their interests in managing marine and coastal resources collide with the interests of NGOs who are engaged with marine and coastal conservation in Berau.

The Berau Marine Conservation Area (Berau MCA) was established in 2005. The process was initiated by international and national environmental NGOs represented by the TNC-WWF Indonesia collaboration. The model of this MCA was based on co-management between the TNC-WWF Indonesia and the district government in the so-called Joint Program. Mostly the programs developed by the Joint Program underlined the need to support the sustainable use of the Berau marine ecosystem without targeting any specific species. Berau waters are important to conservationists because of their high biodiversity value, their importance in migration routes for marine mammals and as mating, feeding, nesting and nursing grounds of sea turtles in the so-called Coral Triangle (Interview with ex-project leader of TNC-WWF Joint Program, Bogor, August 2010)^3^. However, even though the NGOs were not specifically targeting sea turtle, as they used a spatial rather than a species oriented approach to conservation, most of the regulations issued by the district government regarded sea turtle protection. At the same time, the environmentalists strongly opposed sea turtle egg exploitation by the district government through their auctioning (*lelang*) of the right to control sea turtle egg collection on the islands of Berau by private entrepreneurs (see below).

Evidently, the exploitation of the turtle eggs has a long history is Berau. It dates back to the colonial era when the Dutch resident gave permits to local elite families to continue to harvest turtle eggs according to their pre-colonial arrangements, but on the condition that a percentage was earned by the government (Krom 1940; Kusumawati 2014: 65–70). During the New Order era (1965–1998) sea turtle eggs were the only natural resources that could be autonomously managed by local people. Recently, in the era of decentralization, sea turtle eggs have again become one of the sources of district revenue, and are highly valued culturally as well as economically by the Berau government and the community. In the local and regional markets, because of their high quality, the turtle eggs from Berau are quite expensive (10,000 IDR/egg in 2010). Hence, as stated by one of the Joint Program officers, ordinary and poorer people (*masyarakat menengah ke bawah*) could not afford to buy sea turtle eggs. Only the more affluent people could afford it: usually entrepreneurs or government officials. Moreover, government officials enjoy the privilege of receiving a share of the sea turtle eggs from the auction (*lelang*) winner as an informal ‘gift’ to maintain good relationships with the authorities (Interview with ex-project leader of TNC-WWF Joint Program, Bogor, August 2010).

Based on Government Regulation No. 7/1999 on plant and animal preservation, it is forbidden to use any part of the body of the sea turtle, including the eggs. Nevertheless, using the argument that the management of sea turtle eggs is part of Berau’s history and that there would still remain many sea turtles nesting on the shores of the small islands in the Berau Delta, the district government wanted to maintain control over the exploitation of sea turtle eggs and continued the auction of rights to exploit sea turtle nests on islands of Berau district^4^. The district government believed that they could sustainably manage the number of sea turtle eggs traded for the sake of district revenues. Their interpretation of ‘management’ – with a clear economic objective – conflicted with that of the Joint Program who saw the management of sea turtles solely in terms of conservation for the sake of the animals’ future existence. The environmental NGOs in the Joint Program feared that, if the district continued to ‘manage’ sea turtles their way, their numbers in the Berau waters would decrease considerably over the next ten to fifteen years. The Joint Program concluded that the district government was after all not committed to genuine conservation, though when they first introduced the idea, the NGOs received full support from the district officers involved.

The conservation program aiming at improved coastal resources governance in East Kalimantan was not set up as a community-driven development project. The international environmental NGOs (TNC/WWF) did indeed hope for a more effective intervention by bringing resource governance ‘closer to the ground’, choosing a decentralized approach to marine conservation by directly addressing local governance actors (Kusumawati and Visser 2014). District government agencies and local NGOs were selected as members of a Joint Program decision-making body. But the local economic elite was excluded, ignoring their environmental knowledge, historical rights and cultural values regarding resource appropriation and distribution among their dependents. However, accusing the aquaculture bosses and marine resources traders of frustrating the effectiveness of coastal management projects makes little sense, and may even be regarded as demonstrating foreigners’ arrogance in failing to acknowledge the multiplicity of obligations, rights and duties of these patrons or bosses as leaders of coastal communities.

One high profile member of the Berau entrepreneurial elite was Haji Penyu^5^. Haji Penyu was the last and most powerful *pachter* (Dutch: lease holder) of Berau before the exploitation of sea turtle eggs was completely banned in 2005/2006. Since colonial times, and even before in 1880, there had been customary constraints in place on the proportion of eggs that were taken by collectors. These regulations became government mandated (Krom 1940: 84–85; Kusumawati 2014: 66–68). For example, it was decreed that no eggs were to be harvested during the months of November and December. Further, as of 1936 the leaseholder was obliged by legal contract to deliver 200 young turtles at the end of the lease-year on behalf of the government, which were to be released into the sea under supervision of the sub-district’s head of Derawan Island (Krom 1940: 85).

Haji Penyu’s family held a historical position as lease-holder. To the Berau people, he served both as an informal and a formal leader. Formally he was head of Derawan village and he was a businessman. His main business was the sea turtle egg trade. With the money collected from this business, he spread his wings by investing in the construction sector. About sea turtle conservation, he had his own opinions. According to him, the sea turtle population was still in good condition. He still found sea turtles swimming around the lodges at Derawan Island, even though the sea turtles on Derawan Island had not been officially protected, at least not until 2002. Moreover, he stated that Berau was widely known as a producer of sea turtle eggs for regional markets, so the claim made by environmentalists that the sea turtle was an endangered species in his eyes only served the interests of the scientists who worked with the NGOs to justify the MCA project in Berau.

Haji Penyu was also an important patron and “leading figure for all the clients” (Pelras 2000: 16) outside as well as inside the local political arena*.* He acted as a patron of the Berau government, as was confirmed by the ex-project leader of TNC-WWF Joint Program who acknowledged that: “even though Tanjung Redeb is the capital of Berau, where the district head’s office and the sectoral offices are located, we all know that the government is run from Derawan” (Bogor, August 2010). Haji Penyu had an extensive local and regional network, including his younger brother who was one of the vice-chairs of the Berau parliament (DPR). His uncle had served as the head of the department of Fisheries of Berau (DPK) from 2009 to 2012. Before his uncle became the head of the department, this institution was actively involved in monitoring and surveillance activities of the Berau waters together with other Joint Program members. Clearly, Haji Penyu’s institutional alliances over time influenced his political attitude regarding conservation. Commenting on this shift of interest away from conservation by DPK, one of its officers stated:

DPK was involved in monitoring and surveillance activities. But when the head of DPK was replaced [in 2009], we were not permitted anymore to join the monitoring. The Joint Program kept asking us to join their activities, but we were reluctant to join them. We are under the authority of the district government, so we have to obey them. A lot of eyes are upon us. Whatever we do we are always wrong. (DPK officer, Tanjung Redeb, Berau, October 2009)

The replacement of the head of DPK proves the district government’s position regarding the management of sea turtle eggs. DPK heads before him supported marine conservation in Berau. The head of DPK of 2000–2005 supported the idea of the NGOs to promote the sustainable management of marine and coastal resources (Interview with former head of DPK 2000–2005) and also his successor who chaired the DPK from 2005 to 2007. Before 2006, when the district issued the regulation on the full protection of sea turtles, the district government (through DPK) set aside some of its budget for sea turtle conservation activities. The budget came from the share of the taxes generated by the district government from the sea turtle egg trade. When the trade in eggs was declared illegal in the district, DPK lost control over the conservation of the sea turtles.

The only way the Marine and Fisheries Agency knew of ‘doing conservation’ was by hatching the eggs that they received from the leaseholder or *pachter* (Haji Penyu). So, after 2006, because of the invisibility of the trade, if they bought eggs, it meant that they would support an illegal activity according to national and international laws and regulations. Their dilemma was also aggravated by the demise of the legal government–*pachter* contractual relationship; hence DPK was not inclined to continue doing anything to ‘conserve’ the sea turtle. Similarly, the Regional Planning Board (BAPPEDA) and the Tourism Agency argued that before 2006 the district was actually more effective in turtle conservation.

Despite the full legal protection of sea turtles, all actors including government officers of DPK, Regional Planning, Tourism, the lease holder and even BKSDA believed that sea turtle eggs encountered at the market in Samarinda were still originating from Berau^6^. They recognized these eggs by looking at their physical appearance; the eggs sold from the Berau coastal area were mainly the eggs of the green turtle, because their eggs are bigger than those of other species. Still, egg sellers in Samarinda insisted that those ‘Berau’ eggs came from Banjarmasin (South Kalimantan) or sometimes from a sea turtle hatchery in Pontianak (West Kalimatan).

Thus, the kinship relationship between the head of the Berau Fisheries department, Haji Penyu, and the vice-chairman of local parliament clearly frustrated the implementation of marine conservation related activities in Berau. They saw the MCA as an obstacle to their family interest in keeping the concession. One of the Joint Program members stated:

[Haji Penyu’s brother] has a position as the vice-chairman who is in charge of environmental affairs, including fisheries. As long as he is in that position, I do not expect that we would give any support [to implement] the MCA, especially for the budgeting, because he is the one who has the power and authority to arrange the budget for these affairs. [Even though] in my opinion, Berau has the financial capacity to set apart a budget for the monitoring of the Berau waters. (Member of Joint Program, Tanjung Redeb, Berau, October 2011)

The Joint Program officers were annoyed when the district government did not want to share the budget for the necessary monitoring activities. One of the Joint Program officers who was in charge of sea turtle conservation management argued that this was because of the interests in local parliament (DPRD) who were supporting sea turtle egg exploitation. He also suspected that the district government of Berau would not contradict the interests of Haji Penyu and his family. Despite this important role of Haji Penyu that affected the implementation of the Berau MCA, the Joint Program did not want to collaborate directly with Haji Penyu. When they invited Haji Penyu to their meetings, he was formally invited in his capacity as one of the village heads of the coastal area of Berau. In other words, he was invited as a passive ‘participant’ who was expected to only receive the Joint Program’s messages instead of being a partner in a dialogue to find a common ground for conservation practices. Moreover, the Joint Program never recognized and valued his position as a patron of his constituency, including the Berau government.

## Discussion and Conclusion

The East Kalimantan case study presented in this paper supports Sidel’s statement (2004) about the contestation and friction between networks of elite families who are well represented in local politics as well as in private sector economic enterprises, but we question the assertion of a high turnover of power. The fluidity of East Kalimantan’s coastal elite membership and involvement seems to relate more to their shifting resources than to their social power over time (Kusumawati and Visser 2014; Obidzinski 2003).

The Berau marine conservation case addresses the problem of global environmental NGOs collaborating with, and including the local political-administrative elite of Berau, yet at the same time excluding the entrepreneurial elite, and particularly an informal leading figure like Haji Penyu. As a patron to both the coastal villagers and the political elite, he is the real elite. Unfortunately, the global environmentalists of TNC-WWF completely ignored the role of Haji Penyu whose family had obtained legitimacy as the historical lease-holder of rights to control the harvesting of sea turtle eggs in Berau waters. Because of the activities of Haji Penyu, the Joint Program positioned him as their rival in protecting the sea turtle, because the Joint Program saw the high value, locally controlled turtle egg trade as a threat to the sustainability of the sea turtle species.

The dilemma arising from the exclusion of the economic elite from policy-making concerning the MCA and sea turtle conservation is twofold. First, the international NGOs deprive themselves of access to existing local knowledge regarding sea turtles’ behavior and presence in the marine space of Berau. Second, the declared illegality of the egg trade makes it become invisible, going ‘under water’ in a sense, which makes protection difficult to enforce for both local government and the environmental NGOs represented in the Joint Program^7^.

This article concurs with Lund and Saito-Jensen (2013) that studies of elite capture should be based on in-depth and longitudinal empirical investigations. But it also acknowledges the fact that project intervention is caught in legal and short-term outcome oriented frames that severely hamper such an approach. Empirical anthropological research may help out here, although scientists differ among themselves about solutions to minimize the risk of elite capture. We have questioned the need to sever the power relationships between patrons and their dependents (Wong 2010; Labonte 2011) in the context of marine and coastal resources governance in Berau, because such a ‘solution’ disregards the multiple social, economic and political sources of a patron’s power. The Berau case clearly shows a local governmental elite who participate in the same network with an economic elite, both making use of the cultural and historical legitimization of their access to power to govern sea turtle egg exploitation (Kusumawati et al. 2013).

Like many other programs on community development which avoid working directly with local patrons out of concern that they would try to dominate and corrupt planning and governance (Platteau 2004; Dasgupta and Beard 2007; Alatas et al. 2013), the Joint Program also failed to actively engage in a dialogue with Haji Penyu in their MCA project in Berau. They avoided collaborating with Haji Penyu as the informal leader and member of the entrepreneurial elite of Berau because of differing values and perceptions of marine and coastal resources governance. However, when the Joint Program ignored the role of Haji Penyu as a prominent member of the political-economic elite of Berau, they indirectly eliminated the chances to collaborate with the members of his network in the implementation of the Berau MCA, including the most powerful members of the local elite who were patrons of a large coastal clientele, including members of the local government. The result was that the Joint Program as a foreign institution had no access to Haji Penyu’s powerful and extensive political-economic network within and beyond Berau district. Exclusion thus mainly served to sustain the claim of the interventionists that the local elite frustrated their project by ‘misusing project funds’, which in this case merely meant acquiring private and public (district government) income through maintaining a market in turtle eggs.

The point here is not the weakness of State legal frameworks. The frameworks certainly are in place and known by the local officials, but decentralization laws provide sufficient discretionary room for the district head and the heads of government agencies to escape from them in order to give priority to their economic interests as members of local patronage networks. Therefore, the crippling of conservation implementation is not sufficiently explained by stating that the problem lies with weak legal frameworks. Local government leaders turned a blind eye to the turtle egg trade since they were clients of Haji Penyu. In their view, the turtle egg trade was legitimate, even if illegal. Haji Penyu developed his patronage networks to the extent that he and his family could control the Berau district government. It started with his brother who became the district parliament member for Berau (DPRD); then his family business became one of the main financial sponsors of one of the district head/vice-district head’s joint candidature. Finally, his uncle became the head of the Berau Department of Fisheries (DKP). This familial network strengthened Haji Penyu’s position vis à vis the NGOs. In other words, the power of the social and political networks of Haji Penyu immobilized DKP – being one of the most important partners of the NGOs for implementing their marine conservation program – in supporting the management of the marine conservation area.

What do we learn from this case study? Elite capture is a label often used by policy makers and scientists alike to address the problem of ineffective project implementation. This case study has shown that successful implementation of the marine conservation project in Berau was not hampered by an absence of rigid NGO agendas or legal frameworks. Both the central and district governments had a series of regulations in place for conservation and the sustainable use of certain resources. The problem lies in the implementation of those rules and regulations. There certainly is no need to create new regulations, but to improve the social-political conditions for implementing and strengthening them. Sustainability of conservation efforts is therefore more a matter of the social-political will to invest in obtaining better knowledge of the networking of local political-administrative and economic elites, particularly the interdependencies of government leaders and officials and economic entrepreneurs who act as their patrons. First, the efforts of the NGOs to create a legal set of regulations to strengthen the implementation of the Berau Marine Conservation Area was of no avail because of their disinterest in getting to know and understand the multiplicity of social, cultural, economic and political conditions of MCA implementation. Secondly, implementation of marine conservation was obstructed because the legal framework was at odds with the interests of the local economic and political elites of the district. On the one hand, key figures in local governmental institutions were not prepared to disclose their involvement in economic networks, including the trade of sea turtle eggs. On the other hand, neither the NGOs nor the district government were prepared to actively include Haji Penyu in the process of implementing their conservation regulations. For example, by engaging in a positive debate about balancing global scientific interests in conservation with locally held views on resource extraction-cum-conservation by monitoring sea turtle nesting and hatchlings.

In other words, assuming local elites’ mismanagement to be a fact, and taking the elite as a single social unit will not succeed in solving the problem, because it overshadows the need to understand the social, economic, cultural and political importance of the patrons’ (*punggawa*) networks involving members of the elite as well as non-elite members of their constituencies. Instead of a generic critique of the role of local elites in capturing project opportunities for their own benefit, policy makers and project interventionists need to reframe and rephrase the issue of elite capture into the question: ‘How to capture the elite?’

The key lesson learned from Berau MCA case is that not only the governmental elite is the key but also the local entrepreneurial elite should be involved. It is not simply a matter of inviting a patron (*punggawa*) like Haji Penyu as a passive participant to conservation policy meetings. Also, his historical and cultural knowledge and interests in the implementation of conservation practices should be seriously considered in order to find an effective combination of local economic interests, values and local knowledge as well as global scientific knowledge. Not only do local communities depend on these patrons, NGOs also need these patrons to access the local power networks. Meanwhile, the economic patrons are part of regional, national and international chains of power and resources that are often beyond the scope of NGO intervention. In other words, inclusion of local economic patrons provides conservation projects with access to local environmental knowledge and resource extraction practices, that – if accepted as legitimate – provide greater transparency of the value chain of turtle eggs, in order to be properly monitored in view of global conservation demands.

Fritzen (2007) warns that many projects may successfully change institutional forms but not the cultural-historical norms shaping the all-pervasive existence of public–private networks of political and economic elites who share common resources and territorial interests. This East Kalimantan case study shows that indeed one important precondition to redress this bias is often missing, namely the acknowledgement by institutional project interventionists of the societal relevance of existing power networks and the recognition of the historical power and empirical knowledge of such local entrepreneurial elites (Kusumawati et al. 2013; Kusumawati and Visser 2014). The international NGOs in Berau did not seem inclined to take *punggawa* seriously as partners in the implementation of their conservation projects. In fact, the NGOs were falling victim to the idea of ‘elite capture’, instead of trying to positively engage or capture not only the governmental but also the economic elite, and to seek opportunities to involve local communities by making use of the socially valued patronage networks they depend on for accessing marine resources and decision-making power.

## Footnotes

1 Ordinary fishers do not figure prominently in the decision-making processes that were the focus of this research, because they depend on the entrepreneurial elite. See also Kusumawati et al. 2013.

2 Redistribution of economic, financial and material benefits among local elites' constituencies is beyond the scope of this article. This subject is dealt with by Steenbergen elsewhere in this volume.

3 Although this practice has a long history in Berau, of course the big difference is that from auctioning rights to turtle egg exploitation and trade, the regency obtained 10-20 % of the revenues (Kusumawati, 2014 Tab. 3.1). Krom (1940) stresses the importance of the exploitation of the “sea turtle islands” for state revenues, adding that “It is important to note that a conservation regulation was already included in the contract between the Dutch district government and the lease-holding entrepreneur who was obliged to forbid any egg collection and trade during the months of November and December”. Also, starting in 1936, it was included in the contract that the lease-holder should deliver to the local government a total of 200 young turtles at the end of his term to be released into the sea under supervision of the sub-district head at Derawan Island. (Krom 1940: 84-85, our translation).

4 In July 2000, the then head of the district responded to the letter written by the Director General of Nature Protection and Conservation of the Ministry of Forestry and Commercial Agriculture regarding the auction of the rights to control the sea turtle islands. The district head of Berau mentioned that Berau would like to maintain the auction despite the letter. The rationale given was that auctioning also contributed to the monitoring of the sea turtle islands. He mentioned that the monitoring done by the BKSDA was not effective, as evidenced by the case of stolen eggs on the two islands controlled by BKSDA. In his response, the district head also mentioned that the purpose of this auction system was to conserve the sea turtle by way of the obliging the lease-holder to put aside 10 % of the eggs of the total production to be conserved.

5 Haji Penyu is not his real name. Penyu means sea turtle in Indonesian.

6 It is hard to find any formal data from the statistical bureau (BPS) related to sea turtle exploitation after 2004. Neither Haji Penyu nor the district marine agency had any responsibility anymore to monitor sea turtle exploitation or conservation. Besides, several informants in Berau said that they had not recently seen any turtle eggs being traded in the local market. But in Samarinda in 2010, it was easy to find sea turtle eggs traded openly on the streets. For the Berau people it was difficult to understand and caused jealousy concerning why others could continue trading turtle eggs, while in Berau they could not because of conservation regulations.

7 Part of the dilemma of the Joint Program partnership was, of course, that the international NGOs as foreign guests of the Indonesian government, had no power to curb rent seeking practices of the local governmental elite receiving ‘gifts’ from their patron, the lease holder.
